# Superior Adsorption and Regenerable Dye Adsorbent Based on Flower-Like Molybdenum Disulfide Nanostructure

**DOI:** 10.1038/srep43599

**Published:** 2017-03-08

**Authors:** Sancan Han, Kerui Liu, Linfeng Hu, Feng Teng, Pingping Yu, Yufang Zhu

**Affiliations:** 1 Department of Materials Science and Engineering, University of ShangHai For Science and Technology, Shanghai, 200093, China; 2 Department of Materials Science, University of Fudan, Shanghai, 200433, China

## Abstract

Herein we report superior dye-adsorption performance for flower-like nanostructure composed of two dimensional (2D) MoS_2_ nanosheets by a facile hydrothermal method, more prominent adsorption of cationic dye compared with anodic dye indicates the dye adsorption performance strongly depends on surface charge of MoS_2_ nanosheets. The adsorption mechanism of dye is analyzed, the kinetic data of dye adsorption fit well with the pseudo-second-order model, meanwhile adsorption capability at different equilibrium concentrations follows Langmuir model, indicating the favorability and feasibility of dye adsorption. The regenerable property for MoS_2_ with full adsorption of dye molecules by using alkaline solution were demonstrated, showing the feasibility of reuse for the MoS_2_, which is promising in its practical water treatment application.

During the past decade, organic dye is serious water pollutant which generally present in different industrial fields, such as leather goods, cosmetics, textile, paper, etc.^1^,^2^,^3^,^4^. Most of them are highly dissolved in aqueous solution and quite toxic, which can cause serious damage to the environment and human beings. Therefore, various techniques for the removal of dye have been developed, such as precipitation^5^, photocatalysis^6^,^7^,^8^ and adsorption^9^,^10^. Nowadays, photocatalysis has received widespread attention, but the photocatalysts may cause secondary pollutants in aqueous solution during the chemical reaction^11^,^12^. Hence, adsorption technique deserves extensive investigation due to its low consumption of energy, simple operation, high efficiency, low cost as well as the wide suitability for diverse dyes^13^,^14^. As most common adsorbents, polymer microspheres, mesoporous SiO_2_, and activated carbon were synthesized to study their adsorption properties^15^,^16^,^17^. Though activated carbon is widely used as an excellent adsorbent due to its large surface area and high adsorption capacity^18^,^19^, its high cost and regeneration difficulties restricted its application seriously. Therefore, it is necessary to develop low cost and regenerable adsorbents to meet the requirements of actual application.

Recently, two dimensional (2D) materials which own large surface areas and abundant active sites have been one of the most extensively studied materials^20^,^21^. As a typical representative, graphene oxide and its derivatives are considered as ideal adsorbents for the treatment of organic dye, heavy metal, and bisphenol from the water, etc.^22^,^23^,^24^. However, the synthetic method of graphene oxide involves in the strong acid/oxidant consumption, and its production efficiency is very low^25^. Recently, molybdenum disulfide (MoS_2_) has attracted particular attention in electronics, photonics, and optoelectronics applications, including hydrogen evolution reaction^26^, field-effect transistor^27^, and photodetectors^28^, due to its unique physical, optical and electrical properties. Moreover, MoS_2_ can be easily synthesized on a large scale with low cost, such as hydrothermal, chemical vapor deposition methods^29^,^30^. As a typical 2D material, MoS_2_ should be an ideal adsorbent. However, the report about its environmental remediation is scarce, hence it is of significant interest to examine the role and mechanism of dye adsorption based on MoS_2_ in detail.

Herein, flower-like MoS_2_ nanosheets was fabricated by a simple hydrothermal process, and then annealed in Ar atmosphere at 400 °C to improve the crystallinity and remove the organic linkers on the surface. The study showed that the prepared MoS_2_ owned superior dye (Rhodamine B, Methylene Blue, Methyl Orange) adsorption behavior. Furthermore, dye adsorption performance and adsorption mechanism are evidenced by a series of different dye adsorption experiments. Interestingly, after being washed by the solution of PH = 14, the MoS_2_ samples recover its superior adsorption ability, exhibiting its regenerable property.

## Results

### Characterization of MoS_2_ samples

**Figure 1:**
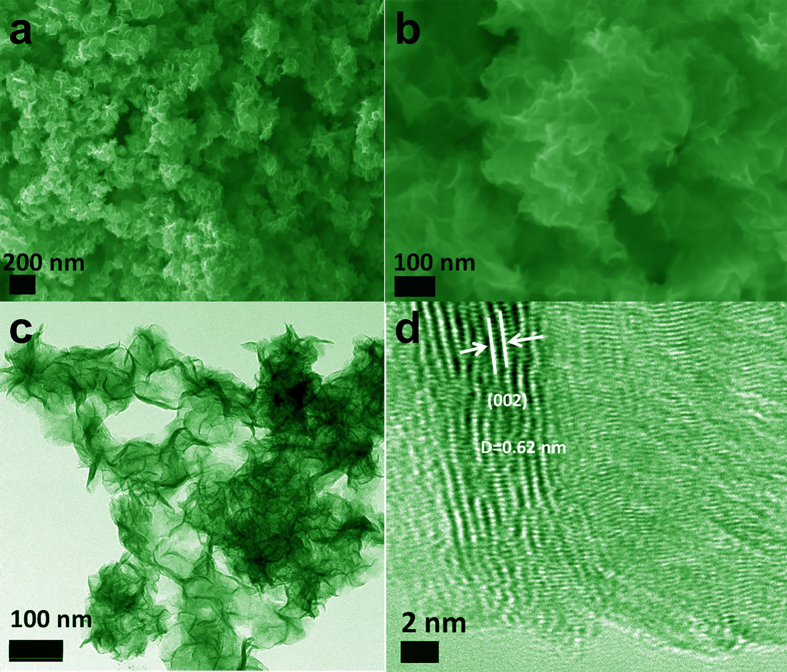
MoS_2_ samples synthesized through a hydrothermal method. (**a**) and (**b**) SEM images of flower-like MoS_2_ nanostructure. (**c**) TEM image and (**d**) HR-TEM image of flower-like MoS_2_ nanostructure.

**Figure 2:**
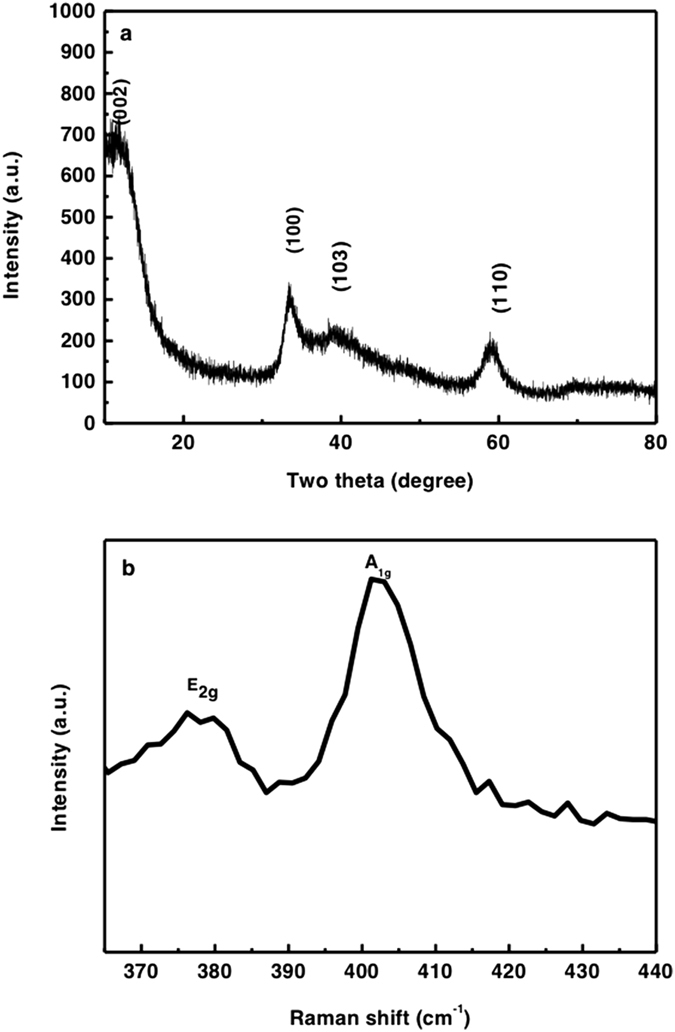
MoS_2_ samples synthesized through a hydrothermal method. (**a**) XRD pattern and (**b**) Raman spectroscopy of the annealled MoS_2_ samples.

The scanning electron microscopy (SEM) and transmission electron microscopy (TEM) images of MoS_2_ architecture annealed in Ar atmosphere at 400 °C are displayed in Fig. 1. Figure 1a,b shows the average size of the MoS_2_ nanostructure is ~200 nm, and such three dimensional flower-like structure owns the large surface area, which is significantly beneficial to the effective adsorption. In Fig. 1d, the high-resolution TEM (HRTEM) image shows that the distinguished lattice spacing is 0.62 nm, which corresponds to the (002) plane of MoS_2_. Moreover, the crystal fringes of (002) plane along the curled edge may indicate the formation of 3–8 layered MoS_2_^31^. Figure 2 shows the X-ray diffraction (XRD) pattern and Raman spectrum of the MoS_2_. From Fig. 2a, all the diffraction peaks can be indexed to hexagonal MoS_2_ phase (JCPDS card No. 37–1492). The peaks at 12.0°, 33.5°, 39.7° and 59.2° can be ascribed to (002), (110), (103) and (110) planes of MoS_2_, respectively. As shown in Fig. 2b, two characteristic Raman active modes of 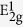 and A_1g_ are located at 377 cm^−1^ and 402 cm^−1^, which associate with the vibration of sulfides in the out-of-plane direction^32^. The big discrepancy between 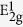 and A_1g_ means the formation of relatively thick MoS_2_ layer, which is accordance with the HRTEM results.

**Figure 3:**
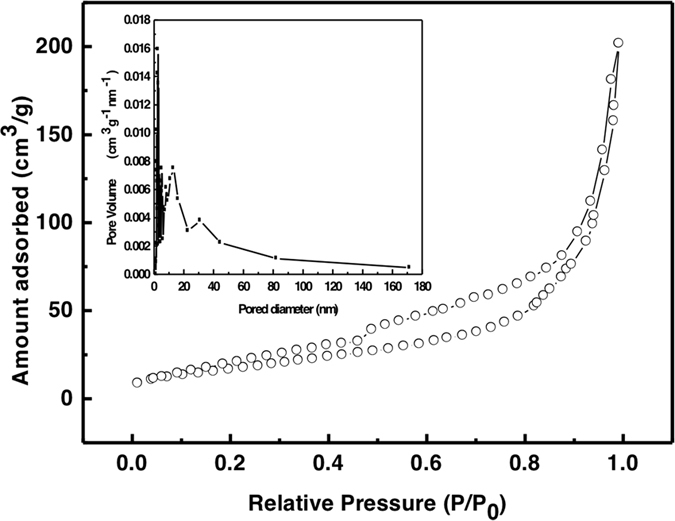
MoS_2_ samples synthesized through a hydrothermal method. N_2_ adsorption−desorption isotherms and the pore size distribution by the Barratt–Joyner–Halenda (BJH) adsorption (inset).

In general, large surface area which provides more active sites is helpful to the diffusion of dye molecules, consequently improving the adsorption capacity during the dye removal process^33^. Herein, the N_2_ adsorption-desorption isotherms and the corresponding Barratt-Joyner-Halenda (BJH) adsorption curve for the obtained MoS_2_ were displayed in Fig. 3. The samples show the type V sorption isotherm with a H3 hysteresis loop, indicating the presence of well-developed mesoporous structure and irregular pores in the samples^34^. The pore size of MoS_2_ calculated by the BJH method ranges from 5 to 20 nm with a broad distribution (inset in Fig. 3). The Brunauer-Emmett-Teller (BET) analysis reveals the surface area (S_BET_) of 63.9 m^2^/g, total pore volume (VT) of 0.31 cm^3^/g, and average pore width (D) of 19.5 nm, as shown in Table 1. A relatively high specific surface area (~64 m^2^ g^−1^) of MoS_2_ can provide more adsorption sites, and the relatively large pore size might facilitate the diffusion of dye molecules.

## Discussion

**Figure 4:**
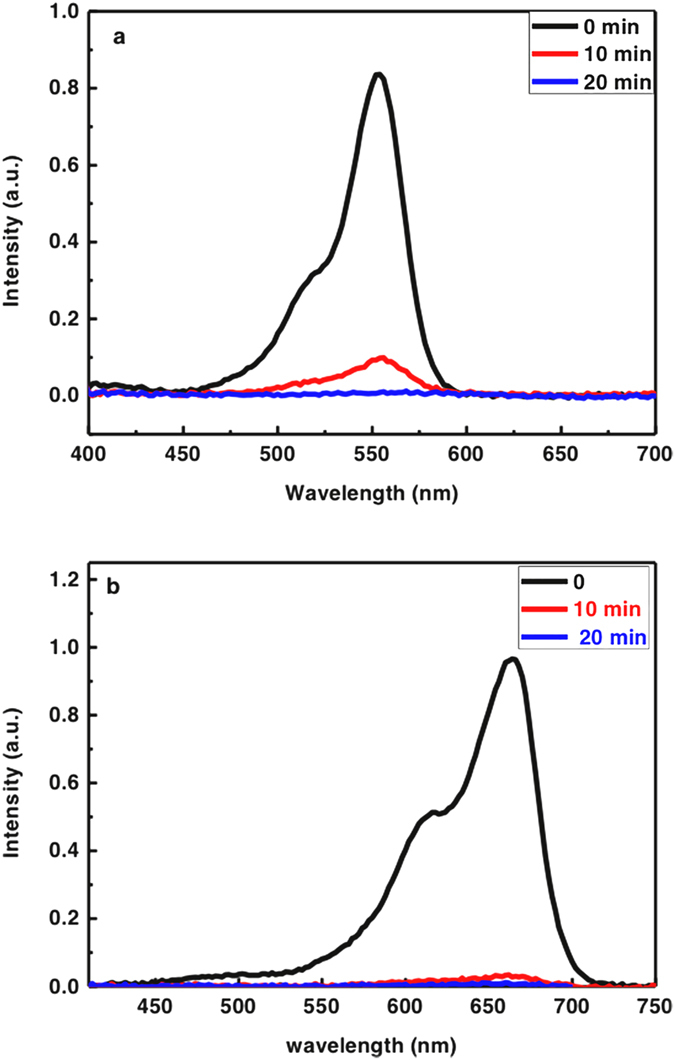
Photocatalytic performances of MoS_2_. Evolution of UV-vis absorption spectra for (**a**) RhB (**b**) MB as a function of time based on 20 mg of MoS_2_ samples.

**Figure 5:**
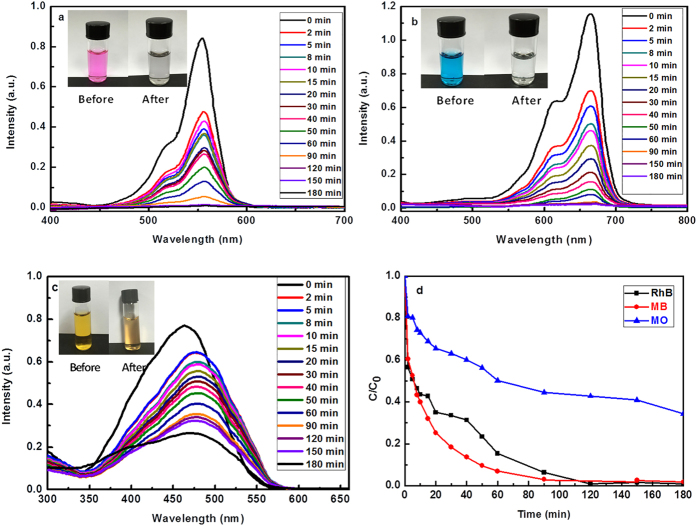
Photocatalytic performances of MoS_2_. Evolution of UV-vis absorption spectra for (**a**) RhB (**b**) MB; (**c**) MO as a function of time (inset: adsorption before (left) and after (right)); (**d**) Removal efficiency of RhB, MB and MO based on 10 mg of MoS2 samples.

As shown in Fig. 4, firstly 20 mg MoS_2_ was taken to confirm the adsorption capability, almost 100% dyes (Rhodamine B (RhB) and Methylene Blue (MB)) were removed within 10 min in our experiment. However, in order to facilitate to investigate the kinetics and isotherms measurements in different industrial dyes (RhB, MB and Methyl Orange (MO)), 10 mg MoS_2_ samples were chosen to slow down the adsorption process. The concentration values of RhB, MB and MO were taken from absorbance at 550 nm, 663 nm, and 464 nm, respectively^35^,^36^,^37^. All the experiments were carried in the dark. Figure 5a–c show the changes of UV-vis absorption spectra after MoS_2_ was added into dye solution, which correspond to the decrease of dye concentration in the solution. Clearly the adsorption process can be divided into two stages in Fig. 5d: the adsorption is very fast due to the high initial dye concentration and unoccupied active adsorption sites at first, then followed by a slow stage, adsorption equilibrium reached. Clearly, the adsorption efficiencies for RhB and MB can achieve almost 100% within 3 h. The color of solution before and after the MoS_2_ adsorption changed from purple, blue to transparency, indicating high adsorption capacity of MoS_2_. In comparison, the adsorption ability of MO is relatively weak, just about 60% of MO was adsorbed within 3 h.

**Figure 6:**
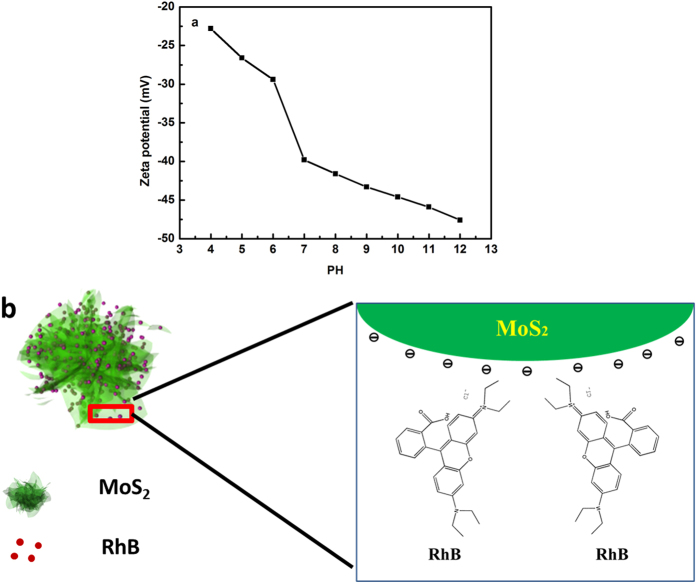
The adsorption mechanism illustration of MoS_2_ samples. (**a**) The Zeta-potential of MoS_2_ at different PH solution; (**b**) The schematic diagram for the adsorption of RhB.

**Figure 7:**
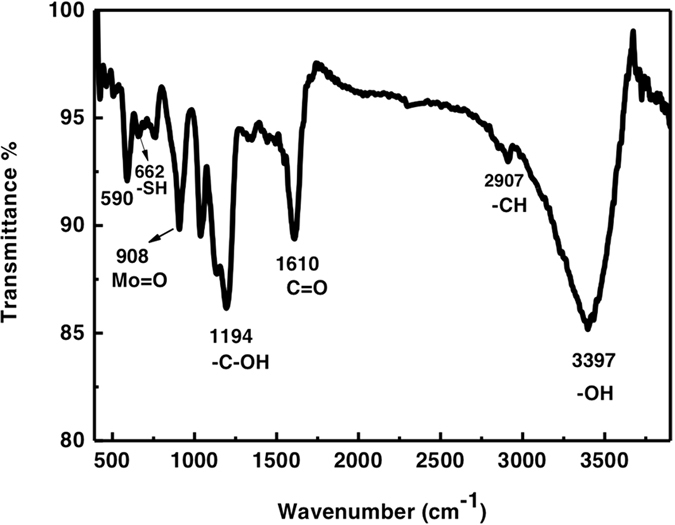
MoS_2_ samples synthesized through a hydrothermal method. The FT-IR spectra of MoS_2_ samples.

To explore the reason why there is huge adsorption difference between cationic and anodic dye, the zeta-potential and FTIR were carried out to study the surface property of MoS_2_. Figure 6a shows that the obtained MoS_2_ has negative surface charge above pH 3, and the zeta potential increases towards alkaline PH, which indicates the abundant acidic sites on MoS_2_ nanosheets^38^. The functional group (-OH, -COOH) maybe responsible for the surface negative charge, which is evidenced by FT-IR spectrum in Fig. 7. Based on above analysis, electrostatic adsorption could be the main factor to selectively adsorb positive charged dye such as RhB, MB compared with negative charged dye MO. Figure 6b shows the schematic diagram for the adsorption of cationic dye RhB, indicating that the obtained MoS_2_ can be superior adsorbent for industrial dye, especially for cationic dye.

**Figure 8:**
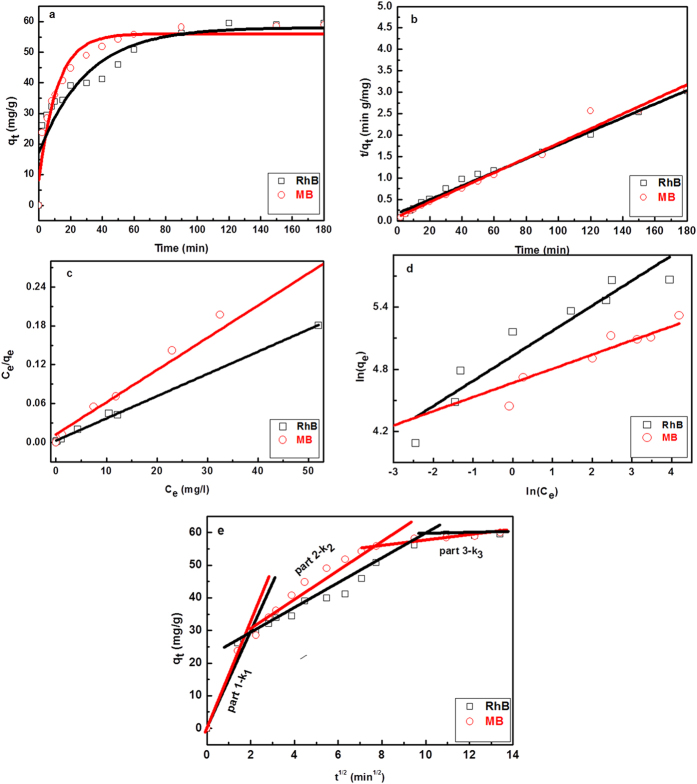
The adsorption mechanism model of dyes based on MoS_2_ samples. (**a**) Pseudo-first-order kinetics model; (**b**) Pseudo-second-order kinetics model; (**c**) Langmuir isotherm model; (**d**) Freundlich isotherm model; (**e**) intra-particle diffusion model for the RhB and MB on the surface of MoS_2_.

In terms of the super-high adsorption capability for cationic dyes, RhB and MB were selected as the indicant reagents to examine the adsorption mechanism of MoS_2_ samples. Herein, the kinetics of these two dye adsorption on MoS_2_ were analyzed by pseudo-first-order model (Eq. 1) and pseudo-second-order model (Eq. 2)^39^,^40^:


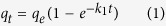



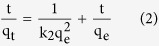


where q_t_ denotes the adsorbed amount at any time t, q_e_ denotes the adsorbed amount at equilibrium. k_1_ and k_2_ denote the rate constant of pseudo-first-order model, pseudo-second-order model, and intra-particle diffusion model, respectively. The fitting results of the models are all shown in Fig. 8a and b, and the calculated data are displayed in Table 2. q_e_ value calculated by the pseudo-first-order model is significantly smaller than the experimental q_e_, and the low values of correlation coefficient (R^2^) of pseudo-first-order model, suggesting the model is not fit to the adsorption process. In contrast, the almost same calculated q_e_ with experimental q_e_, and the high values of R^2^ (>98%) make pseudo-second-order model be more applicable, which implied that the overall rate of the adsorption process was controlled by chemisorption^41^,^42^.

The Langmuir (Eq. 3) and Freundlich isotherm adsorption model (Eq. 4) were used to further determine the adsorption capability of RhB and MB at different equilibrium concentrations^43^,^44^.


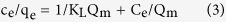



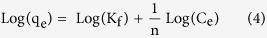


where q_e_ is the adsorbed amount of dye at the equilibrium concentration, C_e_ is the equilibrium solute concentration, Q_m_ is the maximum adsorption capacity and K_L_ is the equilibrium constant of Langmuir. K_f_ and 1/n are Freundlich constants related to adsorption capacity and adsorption intensity.

Figure 8c and d show the fitting results of the Langmuir isotherm adsorption model, and Freundlich isotherm adsorption model. All the values of isotherm constants are given in Table 2. The R^2^ values obtained for Langmuir isotherm adsorption model are greater than that of Freundlich isotherm adsorption model, suggesting that the Langmuir isotherm adsorption model is perfectly fit for adsorption equilibrium of RhB, MB on the MoS_2_ samples.

The basic assumption of Langmuir model is that only one dye molecule could be adsorbed on each adsorption site, and monolayer could form on the surface of the adsorbent, indicating the inter-molecular force and adsorption site decrease with the distance^5^. Hence, the surface of MoS_2_ may have identical adsorption activity, thus providing monolayer dye coverage for MoS_2_ in our experiment. Langmuir dimensionless separation factor R_L_ to determine the favorability and feasibility of adsorption is given in eq. 5:


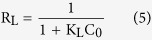


R_L_ indicates the shape of the isotherm, 0 < R_L_ < 1 represent favorable adsorption process and R_L_ > 1 represent the unfavorable adsorption^45^,^46^. As shown in Table 2, all the values of R_L_ are between 0 and 1, suggesting that dye adsorption on the MoS_2_ samples is favorable.

The adsorption process is mainly controlled by two factors: (1) film diffusion, (2) intra-particle (surface or pore) diffusion^47^. And intra-particle diffusion kinetic model was used to determine the rate-controlling step of adsorption based on the Weber–Morris equation (eq. 6).


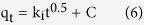


where k_i_ is the intra-particle diffusion rate constant for adsorption at stage, and C is the intercept that represents the boundary layer thickness^47^. In Fig. 8e, the linearized plots of the adsorption amount versus the square root of time were obtained. The straight lines pass through the origin, indicating intra-particle diffusion processes plays a determinative effect in controlling the rate of adsorption. Three different intra-particle diffusion rate constants for the stepwise adsorption influence the rate-limiting steps, as listed in Table 2. The adsorption process can be explained based on the above analysis as follows: (1) The steep slope k_1_ represents the fast adsorption process because of the electrostatic interaction between MoS_2_ and the dye molecules; (2) The slope k_2_ is more gradual, reflecting the dye molecules diffuse into the inner structure of the adsorbent, which is a slowly diffusing process. (3) The flat slope k_3_ is attributed to the adsorption process at equilibrium, where the free path of the MoS_2_ molecules in the pore becomes narrow, and the molecules may also be blocked.

**Figure 9:**
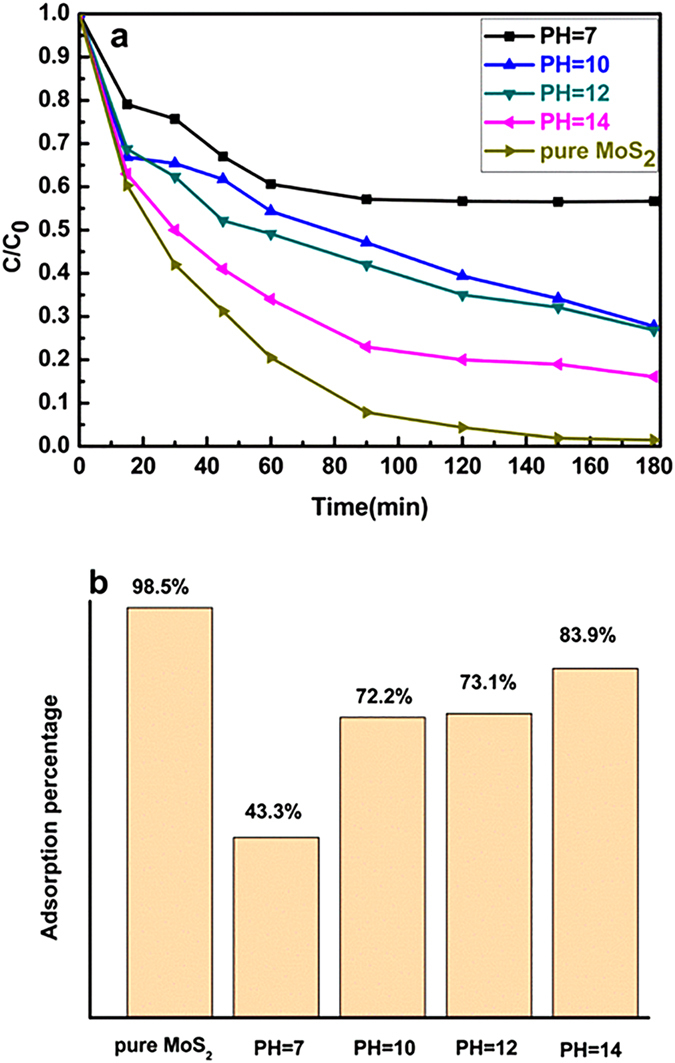
The regenerable performance of MoS_2_ samples. (**a**) Removal efficiency and (**b**) Adsorption percentage of RhB on the surface of MoS_2_ regenerated with different PH solution.

Interestingly, Fig. 9 shows that the adsorption efficiency of RhB for MoS_2_ with full adsorption of RhB molecules washed with alkaline solution is higher than that for MoS_2_ washed with de-ionized water, meaning that the MoS_2_ adsorbents with full adsorption of RhB molecules can be reused easily using different PH alkaline agents to wash, and then the regenerated adsorbents were utilized again to adsorb dye. When PH = 14 alkaline solution was applied, the removal efficiency still remained 83.9% in comparison the efficiency of pure MoS_2_ is 98.5%, meaning that the adsorption ability can be easily recovered by alkaline solution. According to the zeta potential, the excellent desorption performance at alkaline solution can be attributed that excessive OH^−^ ions compete with the activated adsorption sites of the cationic RhB molecules, leading to the desorption of RhB from MoS_2_ through ions exchange^48^. It is confirmed that the feasibility of reuse for the MoS_2_ with full adsorption of RhB molecules by using alkaline solution, which is applicable in its practical water treatment applications.

## Conclusions

In summary, the flower-like MoS_2_ nanosheets have been synthesized successfully by a simple hydrothermal process, which have superior ability to adsorb various dyes and organic pollutants, especially cationic dyes. The obtained MoS_2_ samples own the negative zeta potential, resulting in superior adsorption of cationic dye compared with anodic dye, indicating the dye adsorption performance of MoS_2_ strongly depends on their surface charge. The adsorption mechanism of dye is analyzed, the kinetic data of dye adsorption fit well with the pseudo-second-order model, meanwhile adsorption capability at different equilibrium concentrations follows Langmuir model, indicating the favorability and feasibility of dye adsorption. The excellent reused ability of MoS_2_ has also been confirmed. As a result, the as-synthesized MoS_2_ are promising materials suitable for high-performance pollutant scavenger for water treatment.

## Methods

### Synthesis of MoS_2_

Typically, 0.23 g of Na_2_MoO_4_·2H_2_O (Sinopharm Chemical Reagent Corp.) and 0.8 g of L-cysteine (Sinopharm Chemical Reagent Corp.) were dissolved in 60 mL of deionized (DI) water after stirring for 40 min at room temperature. And then the solution was transferred into a 100 mL Teflon-lined stainless steel autoclave. The autoclave was sealed tightly and heated at 200 °C for 24 h and cooled naturally after the reaction. After cooling naturally, the black precipitates were collected by centrifugation, washed with DI water and ethanol three times, respectively. The samples were dried in vacuum oven at 60 °C for 10 h, and then were annealed in a conventional tube furnace at 400 °C for 2 h in Ar atmosphere.

### Adsorption experiments

All adsorption experiments were carried out in dark and at room temperature. At first, 20 mg MoS_2_ samples were added into 60 mL of the RhB, MB solution with initial concentration of 10 mg/L to confirm the adsorption capability. For the kinetic experiments, 10 mg of MoS_2_ was added to 60 mL of RhB, MB, MO solution with initial concentration of 10 mg/L, then 4 mL of the suspension was taken out at certain time intervals (0–180 min). MoS_2_ samples were separated from the suspension via centrifugation. The measurements of dye concentration were carried out by the Agilent 8453 UV–vis spectrophotometer. For adsorption isotherm measurement, 10 mg of MoS_2_ samples were added to 60 mL of RhB, MB and MO solution with desired concentration (10, 15, 20, 30, 40, 50 and 60 mg/L), then the suspension was stirred for 24 h. For the readsorption experiment, 10 mg MoS_2_ samples with full adsorption of RhB molecules washed by different PH were added to 60 mL 10 mg/l RhB solution. The measurements of RhB, MB and MO were analyzed at the absorbance of 550 nm, 663 nm and 464 nm, respectively.

### Materials characterization

The morphology and composition of the sample were determined by field-emission scanning electron microscopy (FESEM, JSM-6701F), and high-resolution transmission electron microscopy (TECNAI G2 S-TWIN), and X-ray diffraction using Cu Kα radiation (XRD, Bruker D8-A25). The Fourier transformed infrared (FT-IR) spectra and Raman spectra were characterized on Nexus 470 FT-IR spectrometer and Spex 403 Raman spectrometer. The surface area and pore size distribution were performed by nitrogen adsorption-desorption method at 77 K (Micromeritics Tristar ASAP 3000). The ζ-potentials were determined on a Zetasizer Nano (ZS90).

## Additional Information

**How to cite this article:** Han, S. *et al*. Superior Adsorption and Regenerable Dye Adsorbent Based on Flower-Like Molybdenum Disulfide Nanostructure. *Sci. Rep.* **7**, 43599; doi: 10.1038/srep43599 (2017).

**Publisher's note:** Springer Nature remains neutral with regard to jurisdictional claims in published maps and institutional affiliations.

## Figures and Tables

**Table 1:** MoS_2_ samples synthesized through a hydrothermal method.

S_BET_(m^2^/g)	V_T_(cm^3^/g)	D(nm)
63.9	0.31	19.5

Parameters of BET surface area, total pore volume, average pore width for MoS_2_ samples.

**Table 2:** The fitting results of the models.

	RhB	MB
**a**. **First order model**		
K_1_ (g/mg/min)	0.033	0.087
Q_e_ (mg/g)	40.0	47.7
R^2^	0.833	0.936
**b**. **Second order model**		
K_2_ (g/mg/min)	0.0014	0.0030
Q_e_ (mg/g)	62.8	58.4
R^2^	0.992	0.982
**c**. **Langmuir isotherm**		
Q_max_ (mg/g)	291	208
K_L_ (L/g)	1.17	0.40
R_L_	0–1	0–1
R^2^	0.997	0.982
**d**. **Freundlich isotherm**		
K_f_ (mg^1–1/n^.L^1/n^.g^−1^)	138.2	106.5
n	4.14	7.35
R^2^	0.886	0.924
**e**. **Intra**-**particle diffusion**		
k_1_ (min^1/2^ g mg^−1^)	14.8	16.4
k_2_ (min^1/2^ g mg^−1^)	3.89	4.38
k_3_ (min^1/2^ g mg^−1^)	0.05	0.80

(a) First-order kinetics model constant; (b) second-order kinetics model constant; (c) Langmuir isotherm constants; (d) Freundlich isotherm constants; (e) Intra-particle diffusion model constants.
