# Corrigendum: Superior Adsorption and Regenerable Dye Adsorbent Based on Flower-Like Molybdenum Disulfide Nanostructure

**DOI:** 10.1038/srep46887

**Published:** 2017-08-03

**Authors:** Sancan Han, Kerui Liu, Linfeng Hu, Feng Teng, Pingping Yu, Yufang Zhu

Scientific Reports 7: Article number: 43599; 10.1038/srep43599 published online: 03 08 2017; updated: 08 03 2017.

The absorption maximum of MO changes upon adsorption to MoS_2_. Therefore, for calculations of the MO concentration from the absorption spectra shown in Figure 5C of the Article, the authors used λ_max_ ~ 464 nm for time point 0 min and λ_max_ ~ 475 nm for other time points. These details were not included in the Article. To reflect this in the Discussion.

“The concentration values of RhB, MB and MO were taken from absorbance at 550 nm, 663 nm, and 464 nm, respectively”

should read

“At t = 0 min, the concentration values of RhB, MB and MO were taken from absorbance at ~550 nm, ~663 nm and ~ 464 nm. After the adsorption, λ_max_ of ~550 nm, ~663 nm and ~475 nm were used to calculate the concentration of RhB, MB and MO, respectively.”

Additionally, in the Methods section

“The measurements of RhB, MB and MO were analyzed at the absorbance of 550 nm, 663 nm and 464 nm, respectively.”

should read

“The measurements of RhB, MB and MO were analyzed for t = 0 min at the absorbance of 550 nm, 663 nm and 464 nm, and for other time points at the absorbance of 550 nm, 663 nm, and 475 nm, respectively.”

